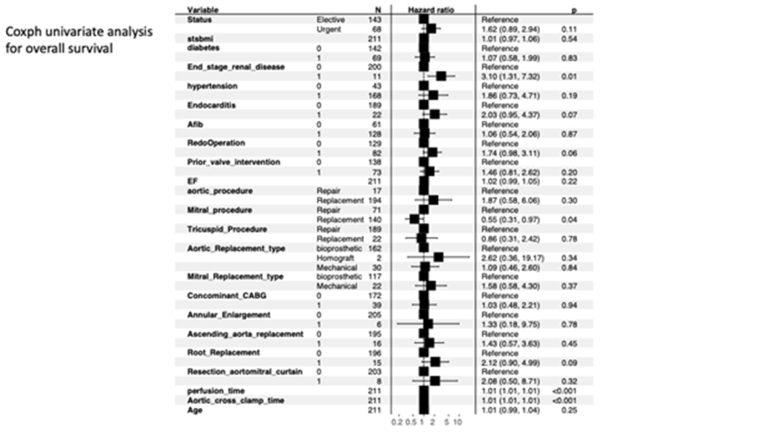# Predictors of Survival After Triple Valve Surgery: A Single Center Analysis

**DOI:** 10.1016/j.atssr.2024.07.021

**Published:** 2024-08-08

**Authors:** Jennie Hocking, John B. Eisenga, Austin Kluis, Kyle A. McCullough, Ghadi Moubarak, J. Michael DiMaio, William Ryan

**Affiliations:** 1 Department of Cardiac Surgery, Baylor Scott and White – The Heart Hospital Plano, Plano, Texas

## Abstract

**Background:**

Triple valve surgery is a complex operation with high perioperative mortality. Limited data exist regarding the predictors of success associated with this surgery. We report our experience.

**Methods:**

A total of 211 sequential patients underwent triple valve surgery at 1 hospital from November 2007 through July 2022. Baseline characteristics, operative details, and mortality outcomes were reviewed.

**Results:**

Eighty-two (38.9%) were redo operations and 68 (32.2%) were urgent. Commonly, aortic (n = 194; 91.4%) and mitral (n = 140; 66.4%) valves were replaced, and the tricuspid valve (n = 189; 89.6%) repaired. Concomitant procedures were done in 28% of patients. Thirty-day mortality was 21 of 211 (9.95%). Kaplan-Meyer survival at 1 and 7 years was 86.0% (interquartile range, 79%-93%) and 64.0% (interquartile range, 55%-74%), respectively. On multivariate regression, end-stage renal disease (hazard ratio, 4.16; *P* = .003) was associated with mortality, and mitral valve replacement (hazard ratio, 0.44; *P* = .009) was associated with improved long-term survival.

**Conclusions:**

Despite the high number of redo and concomitant procedures, we report a 30-day mortality rate under 10% and an 86% 1-year survival. In our series, mitral valve replacement conferred a long-term survival benefit.

## Visual Abstract

**Figure:**
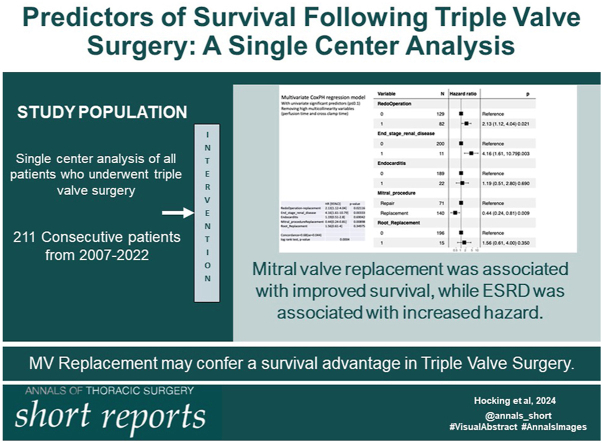

**In Short**

▪ Triple valve surgery can be accomplished with good results in the modern era.
▪ Multivariate analysis noted mitral valve replacement predicted good outcomes, while end-stage renal disease was predictive of poor outcomes.

Triple valve surgery (TVS) is a complex operation historically associated with high perioperative morbidity and mortality.^1^^,^^2^ Despite a growing number of patients undergoing TVS being older and more medically complex, mortality has improved over time.^1^^,^^2^ This trend has been correlated with increased frequency of mitral and tricuspid valve repairs opposed to replacement, which may be due to favorable outcomes in patients undergoing surgical correction of isolated mitral valve (MV) disease.^1^^,^^3^^,^^4^ The translation of these results into patients requiring TVS is logical, although we must remain cognizant that patients requiring TVS are a distinct population and may require different strategies. Reports of good results in patients undergoing replacement of all 3 valves and tricuspid valve replacement being shown to be a poor predictor of late mortality after TVS underscore the unique aspects of TVS opposed to isolated valvular surgery.^5^^,^^6^ Late mortality has been consistently associated with serious medical comorbidities, but otherwise predictors of long-term mortality after TVS are not well described.^1^^,^^3^^,^^5^ This study aims to evaluate modern experiences with TVS to help better understand predictors of long-term outcomes.

## Patients and Methods

### Study Design and Patient Selection

This is a single-center, retrospective analysis of consecutive patients who underwent TVS from November 2007-July 2022. TVS was defined as concurrent replacement or repair of the aortic, mitral, and tricuspid valves. The Society of Thoracic Surgeons Adult Cardiac Surgery Database was queried to identify consecutive patients who underwent TVS at our institution. All identified patients were included in the study. No patients were excluded from analysis. Our institutional medical record was used to corroborate the data from The Society of Thoracic Surgeons Adult Cardiac Surgery Database, to collect further patient information and operative data, and to assess long-term patient outcomes.

This study was approved by our internal institutional review board (IRB#014-209). Informed consent was waived due to the retrospective nature of the study.

### Statistical Methods

Data were tabulated and analyzed using descriptive statistics. Continuous variables were assessed for normality and are presented as mean (SD) or median (interquartile range) as appropriate. Categorical variables are presented as counts and percentages. A *P*-value of <.05 was considered significant for all statistical tests. Comparative testing was performed using Kruskal-Wallis test, χ^2^ test, and *t* test as appropriate. Kaplan-Meier survival analysis was performed to compare overall survival between groups using the log-rank test. Cox proportional hazards regression model was used to assess the association of clinically relevant risk factors with mortality. Risk factors with *P* < .1 in the univariate Cox proportional hazards regression model were used to construct a multivariate Cox proportional hazards regression model. The R statistical software package version 4.0.0 (http://www.r-project.org) was used for statistical analysis.

## Results

A total of 211 patients underwent TVS from 2007-2022. The mean patient age was 68.11 ± 14.37 years, 61.6% (130 of 211) were female, mean body mass index was 27.87 ± 6.61 kg/m^2^, and left ventricular ejection fraction was 52.82% ± 11.82%. Common comorbidities included hypertension (79.6%), atrial fibrillation (67.7%), and diabetes (32.7%). Eighty-two (38.9%) patients had previously undergone cardiac surgery. There was a higher incidence of endocarditis in the patients undergoing reoperation (n = 13, 15.9%) compared with those undergoing primary operation (n = 9, 7.0%) (*P* = .04) (Table 1).

**Table 1:** Baseline Patient Characteristics

Characteristic	Primary Surgery (n = 129)	Redo Operation (n = 82)	Total (N = 211)	*P* Value
Age, y	69.12 ± 14.73	66.54 ± 13.74	68.11 ± 14.37	.09
Body mass index, kg/m^2^	28.19 ± 6.68	27.37 ± 6.51	27.87 ± 6.61	.4
Diabetes	41 (31.8)	28 (34.1)	69 (32.7)	.72
End-stage renal disease	9 (7.0)	2 (2.4)	11 (5.2)	.15
Hypertension	100 (77.5)	68 (82.9)	168 (79.6)	.34
Endocarditis	9 (7.0)	13 (15.9)	22 (10.4)	.04
Atrial fibrillation	72 (63.2)	56 (74.7)	128 (67.7)	.1
Ejection fraction, %	51.43 ± 12.46	55.01 ± 10.42	52.82 ± 11.82	.08

Values are presented as mean ± SD or n (%).

Elective operations accounted for 67.8% (143 of 211) of cases and urgent cases accounted for 32.2% (68 of 211) of cases (*P* = .09). Most commonly, the aortic (n = 194; 91.4%) and mitral (n = 140; 66.4%) valves were replaced while the tricuspid valve (n = 189; 89.6%) was repaired. A bioprosthetic valve was utilized in 162 (83.5%) aortic, 117 (84.2%) mitral, and 22 (100.0%) tricuspid replacements. Concomitant procedures were performed in 59 (28%) patients. In redo operations, the cardiopulmonary bypass (260.07 ± 83.10 minutes) and aortic cross-clamp (182.79 ±5 9.38 minutes) times were significantly longer than in primary operations (230.28 ± 79.17 and 167.53 ± 54.68 minutes, respectively), *P* < .001 (Table 2).

**Table 2:** Operative Characteristics in Patients Undergoing Triple Valve Surgery

Status	Primary (n = 129)	Redo (n = 82)	Total (N = 211)	*P* Value
Elective	93 (72.1)	50 (61.0)	143 (67.8)	.09
Urgent	36 (27.9)	32 (39.0)	68 (32.2)	
Aortic procedure				
Repair	9 (7.0)	8 (9.8)	17 (8.1)	.47
Replacement	120 (93.0)	74 (90.2)	194 (91.9)	
Bioprosthetic	102 (85.0)	60 (81.1)	162 (83.5)	.76
Mechanical	18 (15.0)	12 (16.2)	30 (15.5)	
Homograft	0 (0.0)	2 (2.7)	2 (1.0)	
Mitral procedure				
Repair	52 (40.3)	19 (23.2)	71 (33.6)	.01
Replacement	77 (59.7)	63 (76.8)	140 (66.4)	
Bioprosthetic	67 (88.2)	50 (79.4)	117 (84.2)	.15
Mechanical	9 (11.8)	13 (20.6)	22 (15.8)	
Tricuspid procedure				
Repair	121 (93.8)	68 (82.9)	189 (89.6)	.01
Replacement	8 (6.2)	14 (17.1)	22 (10.4)	
Bioprosthetic	8 (100.0)	14 (100.0)	22 (100.0)	
Concomitant operation	38 (29.5)	21 (25.6)	59 (28.0)	
CABG	29 (22.5)	10 (12.2)	39 (18.5)	
Annular enlargement	3 (2.3)	3 (3.7)	6 (2.8)	
Ascending aorta replacement	7 (5.4)	9 (11.0)	16 (7.6)	
Root replacement	7 (5.4)	8 (9.8)	15 (7.1)	
Resection of aortomitral curtain	5 (3.9)	3 (3.7)	8 (3.8)	
Perfusion time, min	211.35 ± 70.60	260.07 ± 83.10	230.28 ± 79.17	<.001
Aortic cross clamp time, min	157.83 ± 49.30	182.79 ± 59.38	167.53 ± 54.68	<.001

Values are presented as n (%) or mean ± SD.

CABG, coronary artery bypass grafting.

Thirty-day mortality was 21 of 211 (9.95%). Reoperative cases accounted for 15 (7.1%) deaths, while primary operations accounted for 6 (2.8%) deaths. Unadjusted perioperative death among patients who underwent MV repair (n = 7 of 71; 9.85%) was similar to those who underwent MV replacement (n = 14 of 140; 10%) (*P* = .0.97). Mortality was similar in those who underwent tricuspid valve repair (n = 17 of 189; 9%) compared to replacement (n = 4 of 22; 18.2%) (*P* = .17). Implantation of a bioprosthetic valve was associated with early unadjusted mortality benefit for both aortic and mitral valves compared with mechanical valves: 3.41% (4 of 117) vs 45% (10 of 22), respectively; *P* < .001 (Table 3).

**Table 3:** Outcomes at 30 Days as Assessed by Procedural Characteristics

Outcome	Alive (n = 190)	Dead (n = 21)	*P* Value
Primary operation	184	6	<.001
Redo operation	67	15	
Aortic procedure			
Repair	17	0	
Replacement	173	21	
Mechanical	19	11	<.001
Bioprosthetic	153	9	
Mitral procedure			
Repair	64	7	.97
Replacement	126	14	
Bioprosthetic	113	4	<.001
Mechanical	12	10	
Tricuspid procedure			
Replacement	18	4	.17
Repair	172	17	

Values are presented as number of patients.

A total of 8 (3.8%) patients suffered a postoperative cardiovascular accident. In the 178 patients without a preoperative permanent pacemaker (PPM), 39 (21.9%) required a pacemaker postoperatively.

**Supplementary Figure 1:**
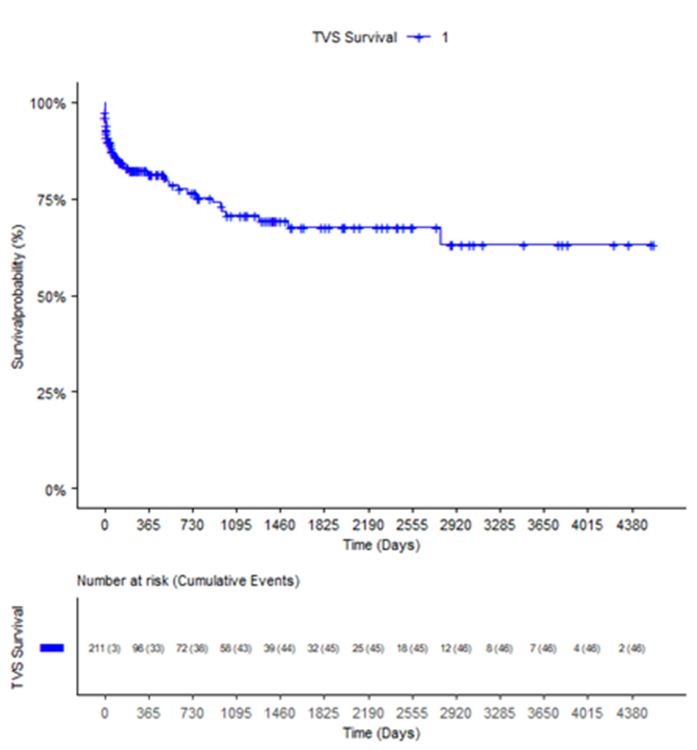


**Supplementary Figure 2:**
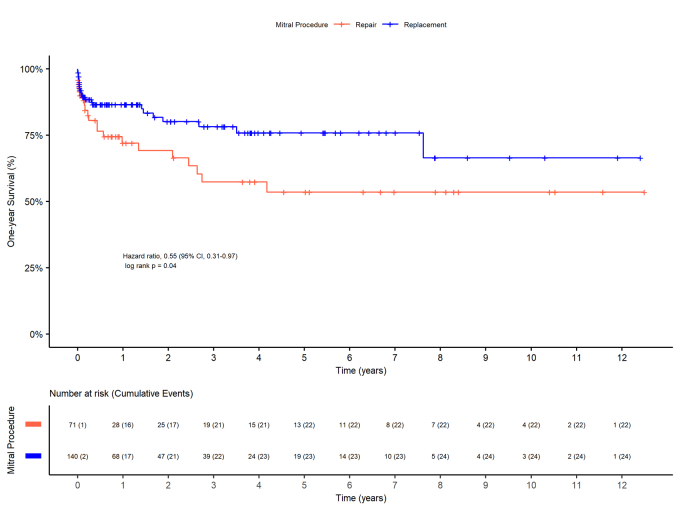


Overall survival was 78.2% (46 of 211) at a mean follow-up of 26.35 ± 35.32 months. Kaplan-Meier survival estimates at 1 and 7 years postoperatively were 86.0% (interquartile range, 79%-93%) and 64.0% (interquartile range, 55%-74%), respectively (Supplemental Figure 1). Significant univariate predictors of long-term mortality included end-stage renal disease (hazard ratio [HR], 3.10; *P* < .01), and increased perfusion time (HR, 1.01; *P* < .01) while MV replacement (HR, 0.55; *P* = .04) conferred improved survival (Supplemental Figures 2, 3). On multivariate regression, both end-stage renal disease (HR, 4.16; *P* = .003) and MV replacement (HR, 0.44; *P* = .009) remained significant predictors of mortality and survival (Figure). Replacement of the tricuspid valve was not associated with long-term mortality (*P* = .78).

**Figure:**
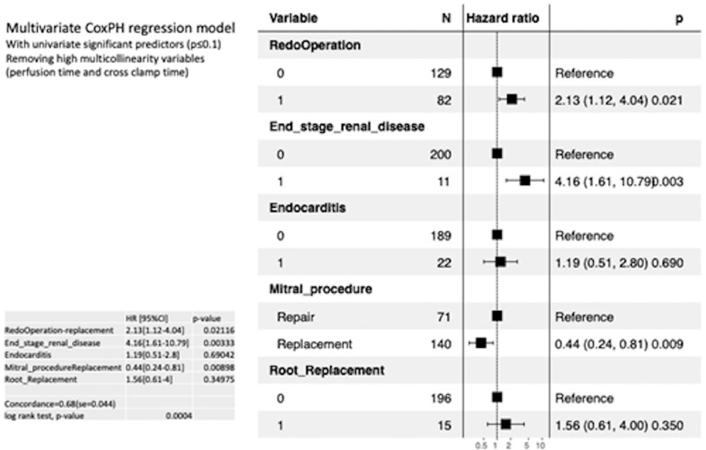
Multivariate analysis of predictors of hazard associated with triple valve surgery. (CoxPH, Cox proportional hazards model.)

## Comment

Despite improvements in perioperative mortality, TVS remains a high-risk surgical procedure, and various factors have been associated with improved outcomes.^1^^,^^3^^,^^5^^,^^6^ This contemporary review of our 13-year experience with TVS at a high-volume dedicated heart and vascular hospital demonstrated the following notable findings: (1) perioperative mortality in patients undergoing primary operation was 2.8%, (2) perioperative mortality for mitral valve repair and replacement were similar, (3) MV replacement conveyed a long-term survival benefit, and (4) end-stage renal disease was a predictor of early mortality.

This study shows excellent primary operative mortality compared with historical studies, and supports modern data demonstrating the safety of TVS.^1^^,^^2^ Our 30-day mortality rate of 9.95% is similar to modern reports, which range from 5.6%-16.1%.^1^^,^^2^^,^^7^ This is notable as we demonstrate a high percentage of concomitant procedures and redo operations, further showing the safety and feasibility of TVS.^1^^,^^2^^,^^7^

Despite reductions in the operative mortality rate, predictors of outcomes are not well established. Across multiple studies, only severe kidney disease has been consistently correlated with decreased long-term survival, which is confirmed by our results.^4^^,^^5^^,^^7^ Other factors such as reoperation, prolonged cardiopulmonary bypass, endocarditis, diabetes, obesity, male sex, and heart failure, have been correlated with poor outcomes in some reports, but this was not observed in our findings.^2^^,^^7^

The impact of valve repair versus replacement on outcomes has been debated. Multiple national database studies have demonstrated significantly increased early survival with repair of the MV, tricuspid valve, or mitral + tricuspid valve.^1^^,^^3^ This finding was thought to possibly be due to shorter operative times, maintenance of ventricular-valvular continuity, or diminished risk of atrioventricular disruption. However, Ohmes and associates^3^ noted that their patients who underwent replacement of all 3 valves had a higher prevalence of malignancy and endocarditis. These factors may have precluded valve repair and may have impacted survival.^3^ In contrast, we found similar 30-day outcomes between patients undergoing mitral or tricuspid valve repair versus replacement, supporting previous studies showing no effect of tricuspid valve management strategy in TVS.^4^^,^^5^ Additionally, after accounting for collinear variables, we found mitral valve replacement conveyed a long-term (7 years) survival benefit. A previous study reported similar findings, noting that although not impacting early outcomes, the odds of late mortality were reduced in those who underwent MV replacement and tricuspid valve repair.^7^ Together, these results suggest that MV replacement may be favorable in the setting of TVS. It is important to note that this study found concomitant coronary artery bypass grafting to be associated with increased perioperative and long-term mortality, whereas our analysis did not.^7^

Additionally, in our cohort, mechanical valve usage was associated with a significantly worse 30-day survival compared with bioprosthetic valves. Although our analysis did not reveal a definite cause, we believe the complications inherent to mechanical valve placement may have played a role.

The exact etiology of our noted survival advantage of MV replacement is unclear. Our patient population may have skewed towards those who would benefit from replacement opposed to repair. Additionally, this was found after accounting for collinear variables, notably, perfusion and aortic cross clamp times. Previous analyses of TVS have reported prolonged cardiopulmonary bypass times to be detrimental to long-term outcomes.^8^ It is plausible that these factors correlate with surgeon efficiency and procedural experience, which may significantly impact outcomes. Further, these operations were performed at a high-volume TVS hospital with multiple high-volume MV surgeons. Increased surgeon volume has been associated with improved outcomes after valve surgery and it is possible that this correlation persists in TVS.^3^

TVS carries a high risk of PPM implantation.^9^ Interestingly, patients requiring PPM implantation have been shown to have similar long-term outcomes to patients who did not require PPM.^10^ By extension, TVS similarly carries a high risk of PPM insertion and our rate of 18.5% is similar to rates with isolated TVS. Future analysis may help determine the full impact of PPM placement after TVS.

### Limitations

This study is limited by its retrospective nature and thus subject to selection bias. Further, our cohort is a heterogenous population undergoing multiple combinations of TVS by multiple surgeons.

### Conclusions

Modern TVS can be accomplished with good results. We noted a significant survival benefit associated with MV replacement vs MV repair, suggesting that MV replacement may be the preferred method for this complex surgery.

## Figures and Tables

**Supplementary Figure 3:**