# Supplementary material for: Predictors of Survival After Triple Valve Surgery: A Single Center Analysis
Source: Ann Thorac Surg Short Rep. 2024 Aug 8;3(1):25–30. doi: 10.1016/j.atssr.2024.07.021 (PMC11910775; doi:10.1016/j.atssr.2024.07.021)
Supplement: Supplementary Material [file mmc1.docx]

**Supplemental Appendix**

Supplemental Figure 1: Overall Kaplan-Meier survival analysis for all patients who underwent triple valve surgery

Supplemental Figure 2: Kaplan-Meier survival analysis of patients who underwent mitral valve repair versus replacement

Supplemental Figure 3: Univariate analysis of predictors of hazard associated with triple valve surgery
